# Multifunctional Loblolly Pine-Derived Superactivated Hydrochar: Effect of Hydrothermal Carbonization on Hydrogen and Electron Storage with Carbon Dioxide and Dye Removal

**DOI:** 10.3390/nano12203575

**Published:** 2022-10-12

**Authors:** Al Ibtida Sultana, Cadianne Chambers, Muzammil M. N. Ahmed, Pavithra Pathirathna, Toufiq Reza

**Affiliations:** Department of Biomedical and Chemical Engineering and Sciences, Florida Institute of Technology, Melbourne, FL 32901, USA

**Keywords:** hydrogen storage, supercapacitor, carbon capture, adsorption isotherm, hydrothermal carbonization, chemical activation

## Abstract

Pore modulation via hydrothermal carbonization (HTC) needs investigation due to its crucial effect on surface that influences its multirole utilization of such ultraporous sorbents in applications of energy storage- hydrogen and capacitive- as well as for pollutant abatement- carbon capture and dye removal. Hence, loblolly pine was hydrothermally carbonized followed by KOH activation to synthesize superactivated hydrochars (SAH). The resulting SAHs had specific surface area (SSA) 1462–1703 m^2^/g, total pore (TPV) and micropore volume (MPV) of 0.62–0.78 cm^3^/g and 0.33–0.49 cm^3^/g, respectively. The SAHs exhibit excellent multifunctional performance with remarkably high atmospheric CO_2_ capture of 145.2 mg/g and high pressure cryogenic H_2_ storage of 54.9 mg/g. The fabricated supercapacitor displayed substantial specific capacitance value of maximum 47.2 Fg^−1^ at 1 A g^−1^ in 6 M KOH and highest MB dye removal of 719.4 mg/g. Higher HTC temperature resulted in increased surface porosity as higher SSA, TPV benefitted H_2_ storage and MB dye removal while superior MPV favored CO_2_ capture. Moderate HTC temperature ensured higher mesopore-to-macropore volume ratio favoring electrochemical performance. Isotherm modelling of the adsorbates was compared using models: Langmuir, Freundlich, Langmuir- Freundlich and Temkin.

## 1. Introduction

By 2040, more than a billion dry ton of biomass will be available annually in the U.S. [1,2] where a significant portion comprises of lignocellulosic biomass. Utilization of such waste biomass for fuel and material development is in dire need, and this has reflected in increasing attention of thermochemical conversions like hydrothermal carbonization (HTC). The solid product of HTC, often called hydrochar, is profoundly studied for applications such as fuel [3,4,5,6,7,8], dye and heavy metal removal [9,10,11]. When it comes to adsorption or storage, the lack of porosity of hydrochar is often deemed as a bottleneck. Therefore, hydrochar is modified to achieve desired favorable porosity, by means of chemical activation that result in porous carbonaceous superactivated hydrochar (SAH) [12]. Such low-cost, ultraporous SAH has recently attracted considerable interest for purposes like capturing greenhouse gases, wastewater treatment and energy storage [13,14,15,16,17,18].

Although volumes of literature attributed to fabrication of various biomass-derived porous carbons used discretely for separate applications, it is imperative to shed light on assessing the applicability of an individual biomass feedstock, like the abundant lignocellulosic biomass, for multiple purposes by tuning the surface porosity development according to its desired application. For the purpose of gas adsorption at high pressure, like H_2_ storage, the adsorbent-adsorbate interaction stems from van der Waals interaction where packing of gas molecules on adsorbent surface in few layers might be impractical, and hence high surface area and total pore volume are found beneficial for enhanced gas uptake especially at higher pressure [18]. On the other hand, for gas adsorption processes at low pressure, like CO_2_ capture, presence of substantial micropores facilitates gas adsorption for adsorbents with high surface area (>500 m^2^/g) as the effect of diffusion in the mesopores becomes wee for adsorption at low gas pressure [19]. For example, Blankenship et al. [20] demonstrated that cigarette-butt derived porous superactivated hydrochar (surface area of 4300 m^2^/g and total pore volume of 2.09 cm^3^/g) primarily promoted high H_2_ storage capacity of 8.1 wt.% excess uptake at 77K and 20 bar. On the other hand, for CO_2_ capture at 1 bar and 25 °C, Sevilla et al. [21] developed superactivated hydrochar (with apparent surface area of 2400 m^2^/g and pore volume of 1.2 cm^3^/g) primarily attributing to the superior CO_2_ capture capacity of 4.4 mmol/g. Similarly, for the purpose of electron storage using supercapacitors hierarchical porous carbon with large specific surface area and reasonable pore structure as the active electrode material promotes high specific capacitance [22,23]. Hierarchical porous carbon works ideally due to the micro (<2 nm), meso (2–50 nm), and macro (>50 nm) pore structures available for rapid ionic motion and effective contact between the electrode and electrolyte [24]. For example, Li et al. [25] presented three-dimensional porous carbon lignin derived supercapacitors, with large mesoporous pore volume and a specific surface area of 1504 m^2^/g, exhibiting excellent specific capacitance of 324 F/g at 0.5 A/g. Additionally, owing to the presence of substantial surface porosity, utilization of such porous carbons for Methylene Blue (MB) dye removal has also been implemented where remarkable performance have been reported with maximum adsorption capacity ranging from 8.96 to as high as 1791 mg/g [26]. These materials make great adsorbents due to high porosity with specific surface area that is capable of trapping the dye molecules from the homogenous solution [27]. Jin et al. [28] presented Chitosan derived three-dimensional porous carbon, performing excellent adsorption of 890.3 mg/g at equilibrium conditions, owing its performance due to increase in active binding sites and suitable pores for diffusion of MB molecules.

Thus, the role of surface porosity, for such various applications of the SAH, is extensively emphasized which is governed by the preprocessing thermochemical conversion conditions [29]. Although pioneered research [30,31] has demonstrated the effect of HTC temperature with altered chemical stability and aromatization in hydrochars, achieving surface porosity modulation by altering HTC temperature, in order to assess its performance on the multirole purposes like H_2_ storage, carbon capture, electron storage, and dye adsorption, is yet to be investigated. Therefore, this study focuses on synthesizing SAH from loblolly pine at various HTC temperatures followed by moderate KOH activation conditions. The SAHs porosity was tailored for various applications, while simultaneously elaborating the effect on other surface properties and hence the subsequent multifunctional role played by the SAH for effective H_2_ storage, CO_2_ capture, electron storage and dye removal purposes. Moreover, the obtained adsorption isotherms were also modelled and compared using Langmuir, Freundlich and Temkin to assess applicability of such models in varied application of gas, liquid, and ionic environments.

## 2. Materials and Methods

### 2.1. Materials Acquired

In this study, biomass used was loblolly pine and it was acquired from Idaho National Lab with particle size ranging from149–595 µm. The chemicals used during activation include KOH and HCl (2 N) which were purchased from Fisher Scientific (Fair Lawn, NJ, USA). Other chemicals used include those for electrode fabrication where carbon black and polyvinylidene difluoride (PVDF) binder were acquired from MTI (Richmond, CA, USA) and N, N-dimethylacetamide (99% pure) (DMAC) was purchased from Acros Organics (Pittsburgh, PA, USA).

### 2.2. Synthesis of SAH from Loblolly Pine

Pine underwent top-down hydrothermal carbonization at 180, 220, and 260 °C for 30 min using 300 mL Parr reactor (Moline, IL, USA), and the procedure of top-down hydrothermal carbonization can be found in detail elsewhere [18]. In short, the biomass to deionized water ratio of 1:10 was loaded in the reactor and the reactor was sealed. The reactor was heated at 5 °C/min until it reached the desired reaction temperature and held the reaction temperature for 30 min. The gaseous products were released in a fume hood. Resulting HTC slurry underwent vacuum filtration using filter paper. Hydrochar underwent thorough washing using deionized water to rinse off the adhered process liquid until it reaches a constant pH. The hydrochar was then dried in oven for 24 h at 105 °C. Hydrochars were then labelled as HT where T reflects HTC temperature.

To fabricate SAH, dried hydrochar underwent activation at 800 °C for 2 h in the presence of N_2_ flowing at 1 L/min. KOH, used as the activation agent, was mixed with hydrochar following a mass ratio (wt.%/wt.%) of KOH: Hydrochar as 2:1. The hydrochar-KOH mixture was then put in alumina crucibles which were then put inside a OTF-1200X-S-HPCVD MTI tube furnace (Richmond, CA, USA) for activation. Once the activation was over, the samples cooled down to room temperature followed by acid wash using HCl acid (2 N) which was followed by a thorough wash with deionized water up to the point neutral pH was reached. The sample was oven dried for 24 h at 105 °C. SAHs were labelled as SAH-A, where A corresponds to the HTC temperature.

### 2.3. Characterization of SAH

Elemental composition was carried out to analyze nitrogen, carbon, sulfur and hydrogen composition (wt.%) by FLASH EA 1112 Series elemental analyzer (Thermo Scientific, Grand Island, NY, USA) where detailed description can be found elsewhere [32]. Oxygen composition was evaluated by method of difference by subtracting the ash content from the sum of C, H, N percentages [33]. Ash was measured in TGA at 575 °C using PerkinElmer TGA 4000 (Waltham, MA, USA).

Appearance of surface morphology was investigated using JEOL JSM 6380LV Scanning Electron Microscope (SEM) (Tokyo, Japan) with the operation parameters: accelerating voltage of 10 and 15 kV, magnification of 1000 and spot size of 40 and 55. To improve the conductivity of the samples’ surface, it was gold coated using Denton Vacuum Desk III vacuum sputter (Moorestown, NJ, USA).

To quantify surface porosity, nitrogen adsorption was employed to determine the BET surface area, pore volume and pore size distribution using Micromeritics HPVA II (Norcross, GA, USA) where the detailed methodology is found elsewhere [32]. In short, nitrogen gas of ultra-high purity was adsorbed for a relative pressure range of 0.009 to 0.995 at 77 K (maintained by means of liquid nitrogen). The generated isotherm data was input to Microactive software where nitrogen adsorption for the relative pressure (P/P_o_) range from 0.05 to 0.35 was used for evaluation of BET surface area where nitrogen adsorption value for P/P_o_ ≈ 0.99 was used to obtain the total pore volume. Moreover, using the nitrogen adsorption isotherms, t-plot analysis was carried out to obtain micropore volume whereas Non-Local Density Functional Theory (NLDFT) was employed using the same Microactive software to obtain the pore size distribution.

Crystallinity of the sample materials were analyzed using X-ray powder diffraction (XRD), employing Bruker AXS X-ray diffraction system (D_2_ Phaser SSD160) (Karlsruhe, Germany). X-ray emission was set with operating voltage of 30 kV and current of 10 mA, respectively, where angular range for data collection was set at 2θ of 5−80° under atmospheric pressure.

Functional group analysis of raw loblolly pine, hydrochars and SAH was carried out by Thermo Scientific Attenuated total reflector (ATR) FTIR (Model: Nicolet iS5, Madison, WI, USA). Operation condition for FTIR analysis were set at: 64 scans, 4 cm^−1^ resolution, and wavenumber range 500–4000 cm^−1^. To quantify total acidic oxygen functional group, Boehm titration was performed. More details of the instrumental analytical methods have been reported elsewhere [34]. The total acidic groups were quantified by means of the following equation:

(1) $$\mu =\frac{V_{{B\left(NaOH\right),}_{s}}-V_{{B\left(NaOH\right),}_{e}}}{M_{s}}\times C_{B}{}_{\left(NaOH\right)}\times 10^{3}$$

where μ is the Total acidic group (μmol/g), *V_B,s_* and *V_B,e_* are the volume of titrant (mL) at end points with and without samples, *C_B_* is the molar concentration of the titrant (mol/L) and *M_s_* is the sample of sample (g).

### 2.4. Gas Adsorption in SAH

H_2_ adsorption was carried out at 77 K whereas N_2_ and CO_2_ adsorption was carried out at 293K, using Micromeritics HPVA II (Norcross, GA, USA). The detailed methodology can be found elsewhere [32]. In short, for each analysis, prior to H_2_ adsorption, the sample was degassed and after that the sample holder with the degassed sample was moved to the analysis port inside a dewar with liquid nitrogen in it to maintain constant temperature of 77 K. Similarly, for N_2_ and CO_2_ adsorption, the sample holder containing the degassed sample was moved to analysis port within Micromeritics ISO Controller (Norcross, GA, USA) with a set temperature of 25 °C. H_2_ pressure steps were set the range 1 to 23 bar whereas for N_2_ and CO_2_ the pressure steps were set in the range of 0.1 to 1.0 bar. Microactive software was used to analyze the generated H_2_ and CO_2_ adsorption data. The unit of total adsorption (n) measured by HPVA II was in mmol/g. This was then converted to N (wt.%), using molecular weight, where the detailed experimental procedure of gas adsorption and data calculation is found elsewhere [35,36].

### 2.5. Electrode Preparation and Testing Using SAH

Fabrication of the working electrode consisted of physically mixing 40 mg of prepared SAH with 5 mg of carbon black. Separately, polyvinylidene difluoride (PVDF) binder was dissolved in N, N-dimethylacetamide (99% pure) (DMAC) using a 1:10 ratio forming a gel-like consistency to transform the mixed carbon materials into a paste. Once the electrode paste was procured, it was transferred into the tip of a borosilicate capillary (outer diameter of 1.0 mm and inner diameter of 0.58 mm) resulting in a mass loading of 0.18 mg. The weight of the capillary was recorded before and after packing to obtain the mass of the electrode material added. The electrical contact was established by inserting a silver wire into the capillary.

The electrochemical performance of SAHs was tested using a three-electrode configuration via cyclic voltammetry (CV) and galvanostatic charge–discharge (GCD) techniques using CHI 660E potentiostat (CH Instruments, Austin, TX, USA) in 6 M KOH. The counter electrode was platinum, and the reference electrode was a saturated calomel electrode. Specific capacitance can be calculated as presented below, where C is the specific capacitance (F/g), I is the current density (A), Δ*t* is the discharge time, m is the mass of the active material in the working electrode (g), and Δ*V* is the potential (*V*) within the discharge time excluding the IR drop.

(2) $$C=\frac{I\times \Delta t}{m\times \Delta V}$$

### 2.6. Methylene Blue Adsorption Using SAH

Batch adsorption experiments were performed and analyzed as reported in detail by Islam et al. [34]. with slight alterations using the produced SAHs with cationic MB dye. To each vial 10 mg of dry SAH was added followed by 10 mL of MB dye solutions of ranging initial concentration of 600–1200 (mg/L). After saturation of the sample is established the final concentration analysis was observed at wavelength 664 nm.

### 2.7. Adsorption Isotherm Models

For the mentioned applications, isotherm modelling and interpretation of suitable adsorption isotherms were investigated. From literature, the frequency of isotherm models used for adsorption processes follows the order of Langmuir > Freundlich > Temkin, Sips, linear, Redlich–Peterson > others [37], based on which, primarily the first three models were applied in this study. Non-Linear methods for determination of model fits were investigated for gas adsorption where linear method was utilized for dye removal and electron storage applications. Origin 2022 software was used to perform non-linear curve fitting for Langmuir, Freundlich and Temkin isotherms using the following equations.

(3) $$q_{e}=\frac{q_{\mathrm{mL}}{\times K}_{L}{\times C}_{e}}{1+\;K_{L}{\times C}_{e}}$$

(4) $$q_{e}=K_{F}{\times C}_{e}{\;}^{\frac{1}{n_{f}}}$$

(5) $$q_{e}=\frac{RT}{B_{T}}\ln K_{T}C_{e}$$

where, for Langmuir q_mL_ is the maximum adsorption capacity (mg/g) and K_L_ is the free energy constant for adsorption (L/mg). For Freundlich *K_F_* is Freundlich capacity factor which is indicative of the relative adsorption capacity of the adsorbent and 1/n_f_ is the Freundlich intensity factor which is a dimensionless constant indicative of the intensity of adsorption. For Temkin R is the gas constant (8.314 J/mol K), T is the absolute temperature (K), B_T_ is the Temkin constant related to the heat of sorption (J/mol) and K_T_ is the Temkin equilibrium constant (L/g) [38,39]. The statistical parameter most commonly considered to evaluate fitting of the results is R^2^ where more than 80% of reviewed references used R^2^ to assess fitness of isotherms [37], which stemmed in utilization of only R^2^ in this study.

## 3. Results

### 3.1. Alteration in SAH Morphology and Functionality with HTC Temperature

The elemental composition of loblolly pine, hydrochars, and superactivated hydrochars are listed in Table S1. It was observed that with HTC temperature increment, C content in the hydrochar increased from 46.2% to as high as 58.43%. From Table S1, it was evident that on hydrothermal carbonization of loblolly pine, carbon enrichment was achieved prior to activation that might aid in enhancing exfoliation during the post-HTC process of KOH activation [40].This also strengthened the importance of HTC to obtain carbon-rich precursors for KOH activation. The percentage of carbon increased from 46.2% in raw loblolly pine to a range of 48.7–58.4% in hydrochars (H180, H220 and H260). Upon activation, further enhancement of C was observed, increasing from 77.1% in SAH180 to 84.2% in SAH260. On the other hand, the H and O content decreased significantly from 4.90–3.50% to 0.06–0.005% and from 42.62–31.65% to 12.93–3.00%, respectively, from hydrochar to SAH. The overall increment of carbon in SAHs reflect aromatic structure dominance whereas the development of aromaticity in the porous adsorbents was also responsible for the removal of H and O from inside of the SAH [41,42,43,44]. Sulfur content was below detection limit (0.024 mg) and was not reported.

**Figure 1:**
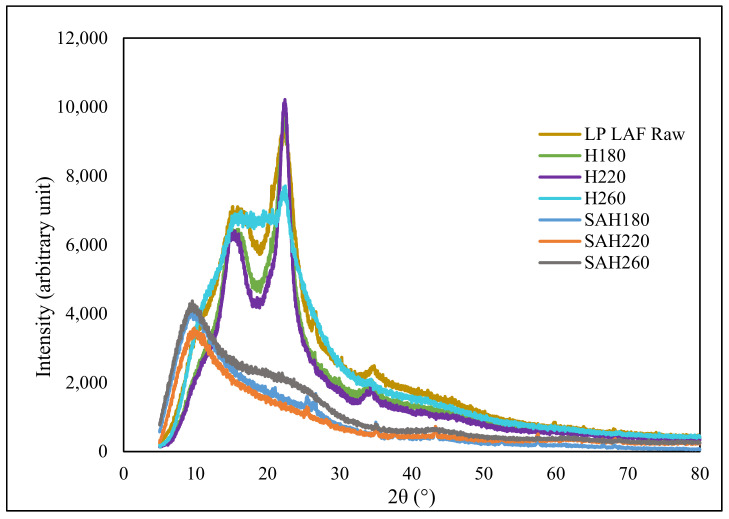
XRD diffractions patterns of loblolly pine biomass, hydrochars (H180, H220, H260) and corresponding activated hydrochars (SAH180, SAH220, SAH260).

The XRD patterns of the SAH and corresponding precursors are illustrated in Figure 1 from where the raw loblolly pine showed diffraction peaks at 2θ~14.7°, 22.2° and 34.6° originating from cellulose [45], while similar peaks were also observed with relatively lower intensity for hydrochars prepared at 180 and 220 °C indicating the preservation of cellulose after HTC. However, the peaks at 2θ ~ 14.7° and 22.2° were replaced in hydrochar prepared at 260 °C with a broad diffraction peak located at 15–21°, which is characteristic of the (002) diffraction pattern of amorphous carbon [45], indicating the onset of cellulose degradation for temperature beyond 220 °C [46]. For SAH, primarily the high intensity diffraction peak is observed in the low angle region (5–15°) that reflect microporosity [47].

Table 1 listed the porosity of SAH and corresponding precursors. Upon HTC, there was slight improvement of surface porosity from 1.6 m^2^/g to 4.8–10.9 m^2^/g in hydrochars, which primarily reflects interparticle voids [48]. Previously, Fang et al. [10] observed similar surface area of 7.8–8.9 m^2^/g in hickory derived hydrochar where HTC was executed at 200–250 °C [10]. With the increase of HTC temperatures, fibrous network in lignocellulosic biomass gets disrupted to form spherical structures with the evaporation of volatile matters [49,50], which might have resulted in higher porosity of hydrochars produced at 260 °C compared to that of 180 and 220 °C in this study. 

**Figure 2:**
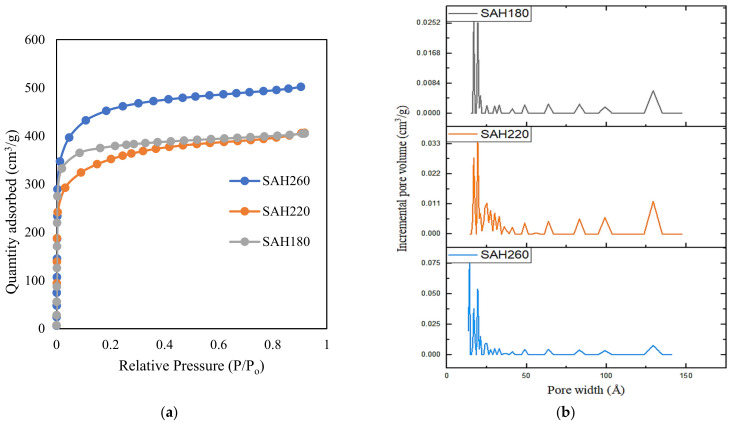
(**a**) Nitrogen adsorption isotherms for superactivated hydrochars SAH180, SAH220, SAH260; (**b**) NLDFT pore size distribution in superactivated hydrochars SAH180, SAH220 and SAH260.

In case of surface porosity of SAH, data from Table 1 illustrates significant improvement of surface porosity in SAH in the form of surface area, total pore volume, and microporosity upon KOH activation of the hydrochars. Analogous finding of significant surface porosity development is also evident in the SEM images in Figure S1. Among SAHs, SAH260 demonstrated the highest porosity where surface area and total pore volume increased by 16.5–30.2% for SAH260 from SAH180 and SAH220 while the microporosity significantly (48.5%) increased in comparison to SAH220. Nitrogen adsorption curves of SAH in Figure 2a follow the shape of type I isotherm that is characteristic of microporous adsorbents by distinctly illustrating sharp nitrogen adsorption at low relative pressure [51]. Moreover, the pore size distribution curve, from Figure 2b, reflects the presence of higher amount of both meso and micro porosity in SAH260 compared to that of SAH180 and SAH220. 

Similar observation was made by Khoshbouy et al. [50] where there was surface area increment from 1475.9 to 1613.9 m^2^/g for porous carbon derived from hydrochars synthesized at 200 °C and 260 °C whereas there was a decrease in surface area to 1428.3 m^2^/g for hydrochar synthesized at 230 °C, concluding the greater impact of hydrochar synthesized at higher HTC temperature on the surface porosity developed in the corresponding SAH. However, hydrochars produced at very high temperature result in more stable hydrochars, which tend to be less reactive towards chemical activation and hence lower pore development [49]. In explanation of the decreased porosity of SAH220 in this study, it might be reasonable to shed light on the possibility of increased particle size of hydrochar H220 to that of H180, as with the increase of HTC temperature aggregation and cross-linking of hydrochar spheres was previously observed by Saha et al. [52]. On the other hand, for the higher HTC temperature of 260 °C the spheres might have lost their uniformity to have burst and broken down to smaller sized particles in hydrochar H260, similarly observed by Saha et al. [52]. Hence, from the discussion above, HTC temperature might have primarily influenced particle size of the hydrochars that lead to the consequent changes in the porosity development of superactivated hydrochars, resulting in the most superior porosity for SAH260.

**Figure 3:**
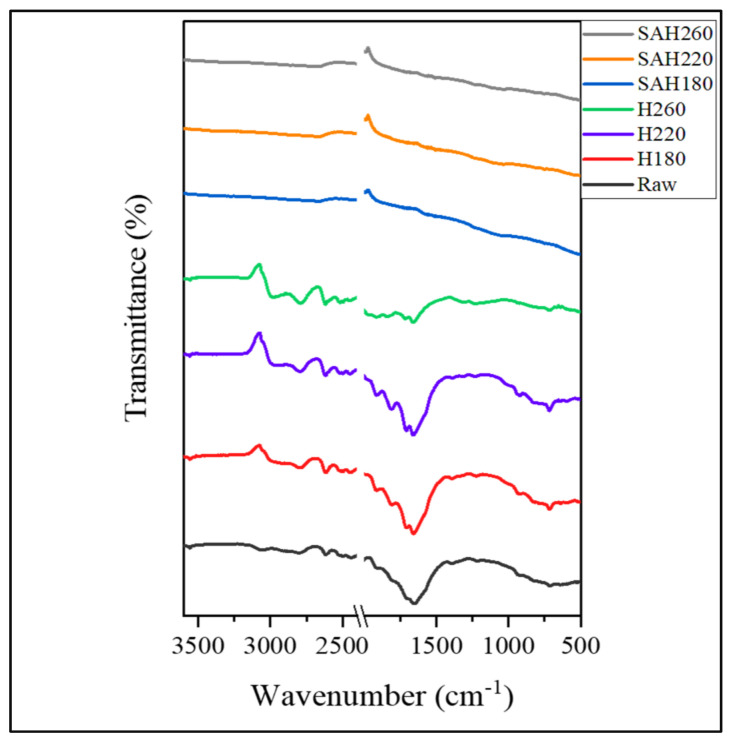
FT−IR spectra of loblolly pine biomass, hydrochars (H180, H220, H260) and corresponding activated hydrochars (SAH180, SAH220, SAH260).

Alteration of surface functionality of SAH with HTC temperature was studied by IR spectra as well as Boehm titration. The IR-spectra of SAH along with raw pine and corresponding hydrochars are shown in Figure 3. Collectively for the super activated hydrochars, SAH180, SAH220, SAH260 there are no peak pronunciation compared to the raw and hydrochar samples as shown in spectra. The reason for this is based on the limitation of the material due to the porous nature of the SAHs as a result the IR light was absorbed disallowing peak functional group detection. As a result, Boehm titration analysis were performed to quantify the available functionality present on the surface of the SAH.

From quantitative analysis on the oxygen surface functional groups performed for the procured SAHs, the total oxygen functional groups (acidic) are listed in Table 2. From the same table, for SAH 180, SAH220, SAH260 total oxygen functional groups (acidic) are 755.8 ± 7.8 μmol/g, 940.4 ± 2.6 μmol/g, and 885.8 ± 1.7 μmol/g while the density of surface functional groups are 0.517 ± 0.005 μmol/m^2^, 0.719 ± 0.002 μmol/m^2^ and 0.520 ± 0.001 μmol/m^2^. Density of functional groups which represents the number of active sites present per unit area (μmol/m^2^) is a contributing feature for adsorption applications. From Boehm titration it is observed that an increase in surface functionality occurred with increase in HTC temperature between temperatures 180–220 °C as result of the carboxylic, lactonic and phenolic organic groups retained from HTC pretreatment followed by a decrease between temperature 220–260 °C. SAH 220 showed the highest total oxygen functional groups as HTC temperature 220 °C maximum retainment of acidic organic groups is experienced while at 260 °C the biomass is susceptible to undergo further dehydration and decarboxylation process causing less retainment of acidic oxygen functional groups. Consequently, SAH 220 presented the highest density of surface functional groups which is owed to its lower BET SSA compared to SAH180 and SAH260, thus allowing higher densification of active functional groups [34].

### 3.2. HTC Affecting SAH Applications

#### 3.2.1. H_2_ Storage in SAH at High Pressure and Cryogenic Temperature

**Figure 4:**
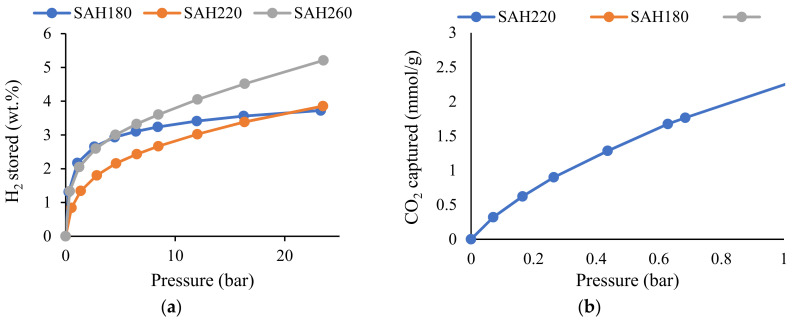
(**a**) H_2_ storage isotherms at 77K up to pressure of 23.5 bar. (**b**) Carbon dioxide capture isotherms at 25 °C up to pressure of 1 bar.

Owing to the large surface area, micropore and total pore volume, SAH were utilized for H_2_ storage and the adsorption isotherms are presented in Figure 4a. It could be perceived that H_2_ uptake increased with the increase of the pressure as the pores tend to be nearly saturated with pressure, leading to greater H_2_ uptake capacity. Moreover, in terms of cryogenic H_2_ storage phenomenon at a comparatively lower pressure (up to 5 bar), hydrogen storage capacity was observed to be equally superior for SAH180 and SAH260. This might be explained by the presence of similar micropore area and micropore volume in both the SAHs (from Table 1) and that is because at 77K the kinetic energy of H_2_ molecules is low which makes it easy for the H_2_ molecules to be adsorbed to the walls of micropores where the H_2_ molecules agglomerate [53]. Moreover, several authors derived positive correlation (R^2^ of 0.911, 0.924 and 0.99941) of micropore volume as well as micropore surface area with cryogenic H_2_ uptake up to 10 bar [53,54,55,56,57]. On the other hand, with the increase of pressure, the interaction between H_2_ and the adsorbate transitions to space filling mechanism [58] where saturation tends to begin where smaller mesopores (2 to 5 nm) become the adsorption site for H_2_ [59,60]. From Figure 4a, it is evident that SAH260 has higher H_2_ storage capacity of 5.21 wt.% (54.94 mg/g) at 23 bar compared to that of 3.72 wt.% and 3.85 wt.%, for SAH180 and SAH220, respectively where the latter SAHs have comparable porosity of 1308–1462 m^2^/g and 0.62–0.63 cm^3^/g. On the contrary, SAH 260 demonstrated to have had a higher surface area of 1703 m^2^/g and pore volume of 0.73 cm^3^/g. Similar observation was made in several other studies where enhanced H_2_ uptake capacity resulted with increment in surface area as well as total pore volume [53,54,55,59,61,62,63,64,65,66,67] where, for example, Wróbel- Iwaniec et al. [55] concluded linear correlation of surface area (R^2^ of 0.94) and total pore volume (R^2^ of 0.92) with the cryogenic high pressure H_2_ uptake capacity. Moreover, the authors [55] also achieved 5.61 wt.% H_2_ storage capacity at 40 bar for chitosan-based activated carbon where the surface area was 3066 m^2^/g, total pore volume was 1.38 cm^3^/g and micropore volume was 1.11 cm^3^/g. Besides, in this study, H_2_ storage was favorably the highest (5.21 wt.% at 23 bar) achieved for SAH260 that possessed superior surface area of 1703 m^2^/g, total pore volume of 0.78 cm^3^/g and micropore volume of 0.49 cm^3^/g, simultaneously illustrating the applicability of loblolly pine derived SAH for excellent H_2_ storage capacity where higher hydrothermal carbonization temperature, 260 °C in this case, resulted in favorable meso-and microporosity for better H_2_ storage.

#### 3.2.2. Carbon Capture on SAH at Ambient Condition 

While H_2_ storage in earlier section was achieved in cryogenic condition for high pressure H_2_ gas, another potential application of the porous activated hydrochars is to capture CO_2_ at ambient condition, where the isotherms of SAH180, SAH220 and SAH260 at room temperature up to atmospheric pressure (1 bar) are presented in Figure 4b. From Figure 4b, highest CO_2_ capture capacity of 3.30 mmol/g (145.2 mg/g) was demonstrated by SAH260 followed by 3.05 mmol/g and 2.25 mmol/g, respectively for SAH180 and SAH220, all compared at 1 bar of CO_2_ pressure. 

Owing to the similar microporosity of SAH180 and SAH260, the CO_2_ adsorption isotherms of those SAHs overlap significantly until a slight difference in the quantity of CO_2_ captured is observed beyond approximately 0.6 bar that might be explained by the superior surface area of 1703 m^2^/g of SAH260. Tao Song et al. [19] observed similar phenomenon and concluded the CO_2_ capture can be increased with the increase of micropore as well as surface area where it was explained to be the dominance of micropores for CO_2_ to be adsorbed as the effect of diffusion in the mesopores gets weakened for adsorbents exceeding BET surface area of 500 m^2^/g. However, it can be summarized as the high adsorption potential of micropores enhance the capture of CO_2_ where the micropores are believed to be responsible for strong capillary forces that facilitates CO_2_ capture at the lower pressure while the mesopores ease the access of pores for CO_2_ to get captured that might explain the primary necessity of microporosity for the availability of more capture sites which could be filled to a higher degree, owing to its stronger adsorption potentials [68,69,70]. A pioneering study conducted by Gogotsi et al. [71] derived positive correlation (R^2^ of 0.9699) between the CO_2_ captured at 25 °C and 1 bar with the pore volume corresponding to those pores smaller than 1.5 nm. On the contrary, a largely scattered linear relationship of surface area with CO_2_ capture at 25 °C and 1 bar was observed in the same study [71] where despite the scattered linear trend between the BET SSA and CO_2_ uptake capacity, higher BET SSA alone did not guarantee superior CO_2_ capture capacity. From this study, it was found that at CO_2_ pressure of 1 bar, SAH260 with the higher CO_2_ capture capacity of 3.3 mmol/g (145.2 mg/g) also displayed superior capture compared to other porous carbonaceous adsorbents with similar porosity in other studies [72,73,74]. For example, Serafin et al. [74] observed CO_2_ capture amount of 2.0–3.25 mmol/g, at 25 °C and 1 bar, for Lloyd, Mistletoe leaves and branches, Kiwi fruit peel and sugar beet pulp-derived activated carbons with surface area of 1263–1699 m^2^/g and micropore volume of 0.41–0.49 cm^3^/g. Moreover, commercially available activated carbons, like BPL and G-32 H, demonstrated comparatively lower CO_2_ capture at 25 °C and 1 bar of 2.1 and 2.5 mmol/g, respectively [72,73]. Hence, SAH260 of this study performed advantageously, also demonstrating better CO_2_ capture with increase of hydrothermal carbonization temperature to 260 °C that might be due to the favorably higher microporosity and surface area in SAH derived at higher HTC temperature. Moreover, the excellent CO_2_ uptake capacity of SAH260 is also accompanied by its selective adsorption capability when compared to N_2_ adsorption isotherm, as illustrated in Figure S2 (Available in Supplementary). The initial slope of all the adsorption isotherms for SAH180, SAH220 and SAH260 also supports superior selectivity for CO_2_ adsorption by indicating significantly higher slope in the adsorption isotherm when compared to that of N_2_ adsorption isotherm. Therefore, ultraporous materials developed in this study demonstrate its successful applicability in CO_2_ capture.

#### 3.2.3. Adsorption of Methylene Blue (MB) Dye from Water by SAH

**Figure 5:**
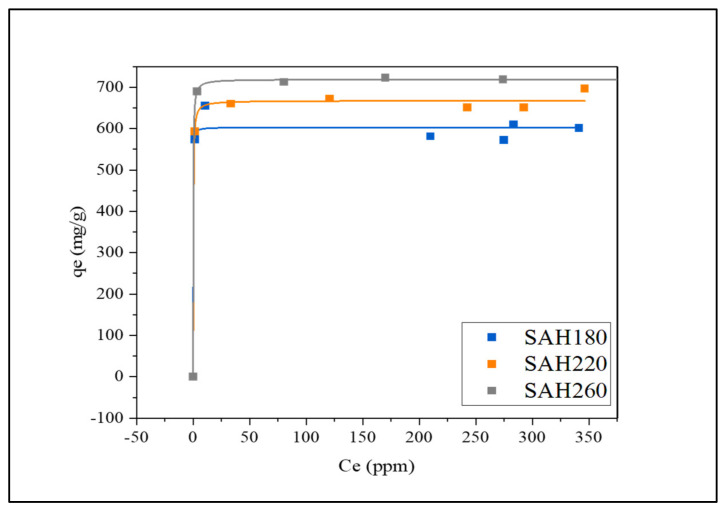
Methylene Blue dye removal by the superactivated hydrochars (SAH180, SAH220, SAH260).

The capture of gas molecules and MB dye are different in application as the different phases allow for different adsorption mechanisms which are later mentioned in detail. In this study, SAH performance for MB dye removal was investigated by varying the initial MB concentration. As shown in Figure 5, for the tested SAH a sharp increase in adsorption capacity is observed with increase initial concentration until saturation is met [28]. Among the investigated SAH the maximum adsorption capacity (Q_max_) was shown for SAH260 followed by SAH220 then SAH180 with corresponding values of 719.4 mg/g, 667.6 mg/g and 603.1 mg/g, respectively. The rapid increase in adsorption capacity is due to the initial dye concentration acts a driving force which overcomes mass transport resistance between the dye and the adsorbent facilitating dye adsorption [75]. On the other hand, the saturation of the adsorption capacity as the initial concentration increases is expressed based on the adsorption isotherm model which is discussed further in the later section. The migration of the dye molecules to the adsorbent is facilitated by the first transport driven by the initial concentration in the liquid phase. Then, second, once in contact with the exterior surface the dye diffuses through the pores of the material granting access to active binding sites for adsorption [75]. Additionally, high surface area improves the adsorption capacity as more adsorbate diffusion into the interior pores is promoted allowing more interaction of the adsorbate to active binding sites within the materials [28].The maximum adsorption capacity values are in close comparison to literature shown in Table S2 (Available in Supplementary document). Bedin et al. [76] presented sucrose spherical carbon derived KOH-activated carbon for MB removal with maximum adsorption capacity of 704.2 mg/g with BET SSA of 1534 m^2^/g, and total pore volume of 0.765 cm^3^/g. In more recent studies, Tran et al. [77] presented carbonaceous hydrochar (BET SSA = 862.2 m^2^/g) derived from coffee husk waste via hydrothermal carbonization followed by KOH chemical activation showing highest MB removal of 415.8 mg/g at 30 °C. For the investigated SAH the BET SSA as well as total pore volume are analogous to literature, proving that high surface area material maximizes adsorption capacity of MB. With increase in HTC temperature SAH180, SAH220, SAH 260 there is an increase in BET SSA, thus an increase in active binding sites for MB [26] therefore showing evidence of pore filling adsorption mech. Thus, showing evidence of pore-filling adsorption mechanism due to higher BET SSA and total pore volume [78,79]. For SAH180 and SAH220 there is a dip in the BET SSA increase trend however the Q_max_ kept the increasing trend with increase in HTC temperature. As mentioned earlier, an increase in the density of functional groups improves the adsorption capacity of the material [34]. SAH220, although containing a lower BET SSA contain active binding sites due to the surface functionality, allowing for a higher Q_max_ than SAH180. This switch in adsorption dominance shows evidence of chemical interactions due to high amount of oxygen containing functional groups as another adsorption mechanism [80,81].However, comparing SAH260 with SAH220 the great difference in BET SSA resulted in higher surface area being the driving influence for Q_max_ rather than surface functionality. 

#### 3.2.4. Electron Storage on SAH

**Figure 6:**
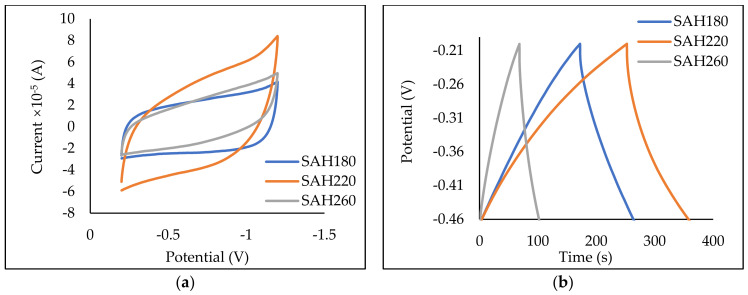

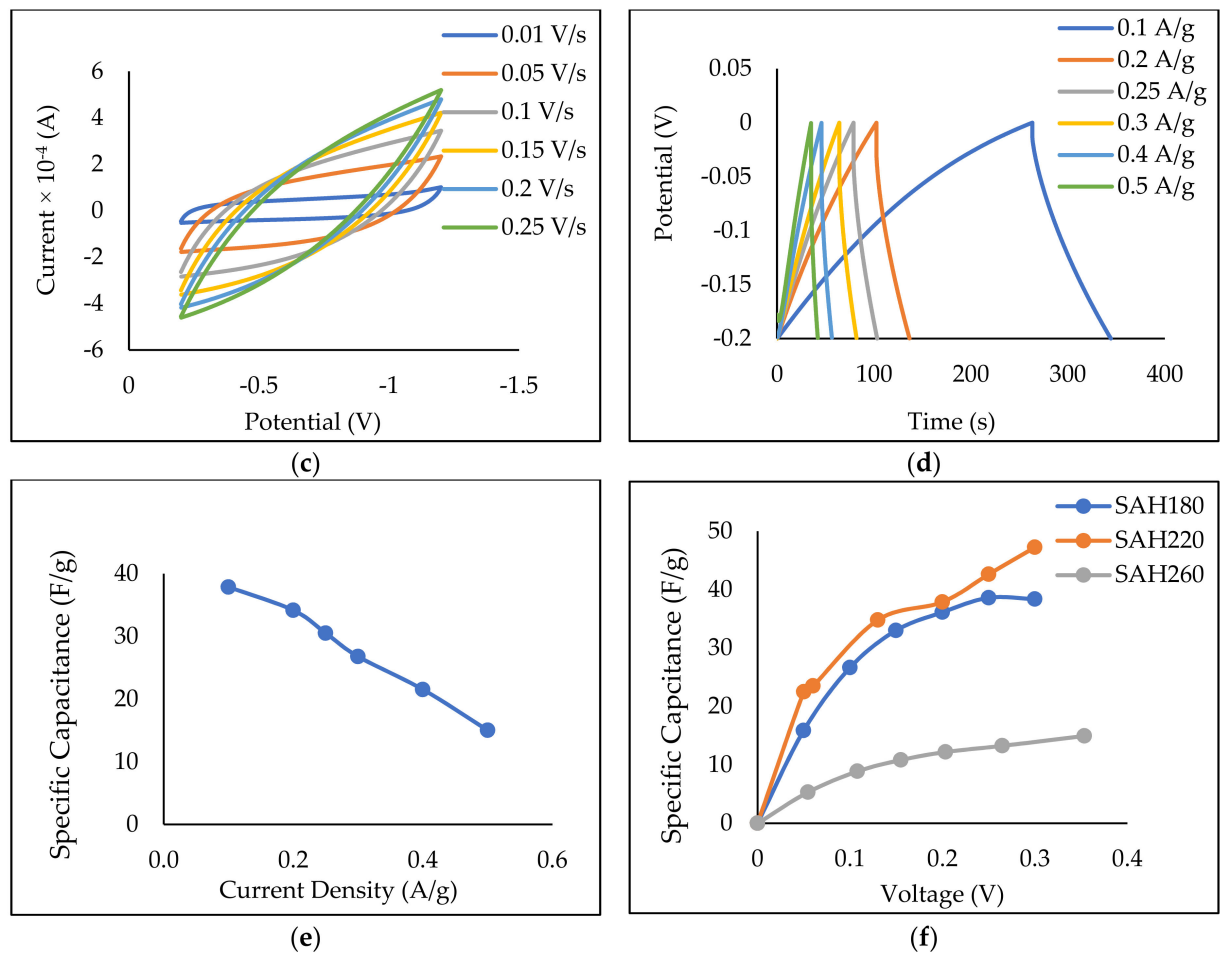
(**a**) Cyclic Voltammograms for SAH 180, SAH220, SAH260, (**b**) Galvanostatic charge and discharge Curve for SAH180, SAH220, SAH260, (**c**) Cyclic Voltammograms for SAH 220 at 0.01–0.25 V/s, (**d**) Galvanostatic Charge−discharge Curve for SAH220 at 0.1−0.5 A/g, (**e**) Specific Capacitance vs. Current Density for SAH220, (**f**) Adsorption Model for SAH180, SAH220, SAH260.

As seen in Figure 6, it is apparent that HTC temperature affects the electrochemical properties of SAH180, SAH220, and SAH260. Cyclic voltammetry experiments performed at the scan rate of 0.01 V/s and potential range (−1.2 to −0.2 V) yielded quasi-rectangular response for all three SAHs (Figure 6a) that are typical of electric double layer capacitor (EDLC) features [82]. The larger the CV curve/rectangular response, the higher the specific capacitance of the active material; thus, SAH 220 produces the largest rectangular area, followed by SAH 180 and then SAH 260. Because SAH220 shows excellent storage capabilities, CVs at varying scan rates (0.01 V/s–0.25 V/s) were performed (Figure 6c). At higher scan rates, the rate of diffusion is higher than the rate of reaction, allowing high specific capacitance; thus, larger rectangular areas in the CVs.

Galvanostatic Charge and Discharge (GCD) experiments were performed at 0.1 A/g with −0.5 V to 0.25 V potential window, with all three systems resulting in quasi-triangular response (Figure 6b). The larger the triangle, the longer the discharge time reflecting in a higher specific capacitance (Cs). SAH220 produced the largest quasi-triangle response, followed by SAH180, then SAH260, and the calculated Cs are 47.23, 38.35, and 13.26 F/g, respectively. This trend in the calculated specific capacitance agrees with the response obtained from the CV tests. As SAH220 showed superior capacitive properties among the three SAHs, further GCD tests were performed, varying current densities (0.1 A/g to 0.5 A/g) (Figure 6d). The deviation from linearity in the GCD curves (Figure 6b) suggests the existence of pseudo-capacitance, resulting from surface oxygen-containing groups, causing redox reactions [25,83]. The gravimetric specific capacitances of SAH 220 as a function of different current densities are depicted in (Figure 6e) indicating that the Cs increases as the current density decreases, which is consistent with previously reported studies [25,83,84,85,86,87,88].

The literature suggests that the driving force for optimizing Cs is the combination of macropore and mesopore volume (V_mame_). Li et al. [25] observed that the mesopores reduce the transmission resistance of the ions while the macropores allow for sufficient electrolyte ions to reach in and out of the active surface, increasing mass transport. However, a large number of macropores will reduce the SSA, negatively affecting capacitance. Therefore, samples with high SSA and V_mame_ favoring mesoporosity will present excellent electrochemical properties [84]. As seen in Table 1, the V_mame_ for SAH180, SAH220 and SAH260 were 0.17 cm^3^/g, 0.29 cm^3^/g, and 0.29 cm^3^/g, respectively, thus, an increase in HTC temperature results in an improvement in the V_mame_. V_mame_ of SAH220 is greater than that of SAH180, resulting in greater Cs for SAH220 than SAH180. Interestingly, the V_mame_ of SAH 220 and SAH260 are the same; however, Cs results reveal that the mesopore and macropore ratios differ. This difference is confirmed by the NLDFT pore size distribution (Figure 2b), as SAH 220 showed higher peaks between pore widths 2.5–13 nm confirming more mesopores compared to SAH 260. Hence, the more mesopores found in SAH 220 allowed for higher Cs. Previously reported studies (Table S3) used electrodes sized at (1–2 cm^2^) with mass loadings (2–10 mg/cm^2^) [25,83,84,86,87,88] thus, more available hierarchal carbon is present for electron storage resulting in higher capacitance. In contrast, our electrodes size is much smaller (0.5 cm^2^); consequently, the mass loading was lower (0.01 mg/cm^2^). However, when these differences in electrode sizes and mass loadings are accounted for, it is evident that our materials have a great potential to be used in supercapacitor applications.

#### 3.2.5. Comparing Adsorption Isotherms of Various Applications

In order to elucidate the sorption mechanism for each application discussed earlier, adsorption isotherms of H_2_, CO_2_, dye, and electron storage were primarily fitted using Temkin, Freundlich, and Langmuir models, where the statistical and model parameters were tabulated in Table 3, considering suitability of a model when R^2^ > 0.99 (rounded to two decimal places). 

Considering the individual applicability of each model for each application, from Table 3, starting with Temkin isotherm model, it assumes adsorption in multi layers [89] and it also assumes that the differential heat of adsorption reduces linearly with surface coverage due to the effect of indirect adsorbate-adsorbent interactions on adsorption [90]. In the Temkin model, the constant b_T_ signifies bonding energy, reflecting the type of adsorbate-adsorbent interaction where heat of adsorption for physisorption is less than 1 kcal mol^−1^ and that of chemisorption is 20–50 kcal mol^−1^ [91]. From Table 3, Temkin model is demonstrated to be suitable for H_2_ adsorption in SAH180, owing to the higher R^2^ value of 0.99681 as the finer experimental data fitting by Temkin model indicated the heterogeneity of the adsorbent surface. In addition, the Temkin model parameter (b_T_) of 0.113 corresponding to adsorption heat of 5.24 kcal mol^−1^ might indicate presence of both physisorption and chemisorption, similarly concluded earlier by Kim et al. [92], where for this study, it might be primarily ascribed to the facilitated affinity of H_2_ in SAH180 due to the comparative absence of oxygen acidic functional groups (OAFG). Several authors [93,94] concluded the presence of such acidic functional groups inducing decreased H_2_ adsorption capacity, where Huang et al. [93] validated that H_2_ adsorption capacity being significantly suppressed on increment of acidic group amounts beyond 0.8 mmol/g, also observed for SAH220 and SAH260 in this study. There was no good fit of Temkin model for CO_2_ capture data in this study. Moreover, Temkin model is inapplicable for applications like MB dye removal and electron storage as this model cannot adequately describe isotherms where adsorption is not fully reversible which translates to its inappropriateness to present aqueous-phase adsorption isotherms [95]. 

Freundlich isotherm model is a widely applied equilibrium model that describes the non-ideal and reversible adsorption, also presumes multilayer adsorption with affinities over the heterogenous surfaces where heat of adsorption decreases exponentially with surface coverage where the stronger binding sites are occupied first [96,97]. Using the model parameters, the Freundlich intensity factor (1/n_F_) is the intensity of adsorption that signifies the type of adsorption where value below 1 implies chemisorption and when the value is greater than 1, it implies cooperative adsorption [98,99]. From Table 3, multilayer adsorption following Freundlich isotherm is observed to be a good fit (R^2^ > 0.99) for H_2_ (except for SAH180), and electron storage as well as CO_2_ capture in all SAH where, the corresponding 1/n_F_ were in the range of 1.42–2.99 for CO_2_ capture as well as electron and H_2_ storage, providing implications of cooperative adsorption. In this study, cooperative adsorption might indicate that the adsorption sites in the superactivated hydrochars were responsible for initiating the propagation of the interactions on the whole frameworks of such porous carbon as well as might be responsible for inter- molecular [100] or inter- ionic interactions [101], where possibility of such adsorption were previously ascribed to the chemical and structural nature of the surface [100,102].

Unlike the Freundlich model, Langmuir isotherm model presumes monolayer adsorption where the thickness of the adsorbed layer is one molecule with no steric hindrance and lateral interactions between the adsorbed molecules and the adsorption sites are finite, localized and identical [103]. Langmuir isotherm corresponds to homogenous adsorption with constant enthalpy of adsorption between each molecule and site of adsorption, reflecting equivalent attraction between the adsorbate and adsorption site [104]. Applying the model, the Langmuir constant is related to adsorption capacity and it can be correlated to surface area and porosity where large surface area and pore volume indicates higher adsorption capacity [39]. From Table 3, MB dye removal solely followed Langmuir isotherm and hence confirming monolayer adsorption which is comparable to literature [28,105,106] whereas utilization of separation factor (R_L_), listed in Table S4 (Available in Supplementary) resulted in the dye adsorption being proven to be favorable with all the R_L_ falling within 0 and 1. On the other hand, from the same table, CO_2_ capture and electron storage are also demonstrated good fit (R^2^ > 0.99) for Langmuir model for all the SAH which shed light on the necessity of analyzing fitting of electron storage and CO_2_ isotherms to Langmuir- Freundlich model, as the evaluated parameters are listed in Table S5 (Available in Supplementary), which is originally a three parameter model deduced to predict heterogeneity of the adsorption system and at low concentration of the adsorbate, the model transforms to Freundlich whereas at higher adsorbate concentration it transforms to Langmuir [107]. For CO_2_ capture, from the same Table S5 (Available in Supplementary), Langmuir- Freundlich model similarly provides fine fit, reflecting both mono and multilayer adsorption of CO_2_, where the parameter, β, lying in the range of 0.803- 0.940, indicates heterogeneity as β ˂ 1 denotes a heterogeneous adsorbate- adsorbent system [108,109]. Findings of Langmuir- Freundlich model for electron storage in the same Table S5 (Available in Supplementary) also depicts both monolayer and multilayer adsorption for the adsorption/desorption mechanism for electron storage. As mentioned, literature has described electron storage model using hierarchical porous carbon to where the macropores act as secondary ion-buffering reservoirs (Langmuir), mesopores provide low resistance pathway (Freundlich) [22]. 

Hence, in summary, Freundlich model primarily demonstrated to be a good fit for cryogenic H_2_ adsorption, indicative of multilayer adsorption of the small H_2_ gas molecules (0.1 nm) whereas Langmuir model was capable of fitting MB dye adsorption data, signifying the presence of monolayer attachment of the large MB dye molecules (0.95 nm) on the microporous superactivated hydrochars investigated in this study. On the other hand, Freundlich-Langmuir isotherm fits were successful in predicting isotherm for intermediately sized CO_2_ molecules (0.33 nm) captured at room conditions which might be possibly due to the presence of both mono- and multi- layered adsorption of CO_2_ in the superactivated hydrochars whereas modelling of electron storage reflecting mono and multi-layer adsorption might be related to the presence of meso and macroporosity in the superactivated hydrochars investigated in this study.

## 4. Conclusions

This study distinctly highlighted the application of abundant loblolly pine, via the application of HTC and KOH activation, for multifunctional utilization in sustainable energy and environmental remediation purposes where the crucial effect of HTC temperature on porosity and consequently on the performance in individual applications was investigated. With HTC temperature increment, 180–260 °C, surface porosity increment was significant where SAH260, owing to high surface area and total pore volume, demonstrated facilitated MB dye removal capacity as well as H_2_ storage whereas microporosity in it greatly assisted in superior CO_2_ capture. Moreover, SAH220 demonstrated excellent electrochemical performances compared to the other two materials due to its high mesopore ratio to macropore volume and surface area. Lastly, the several isotherm modelling fits highlighted the applicability of Freundlich, Langmuir and Freundlich-Langmuir isotherm models to be finer in predicting H_2_, MB dye and electron as well as CO_2_ capture, respectively, reflecting, multilayer, monolayer and combination of multi-mono layer sorption of the respective adsorbates on the superactivated hydrochars synthesized from loblolly pine. 

## Figures and Tables

**Table 1:** Surface porosity characterization of LP, corresponding hydrochars and activated hydrochars.

	Sample	LP	H180	H220	H260	SAH180	SAH220	SAH260
Property								
BET SSA (m^2^/g)		1.6	4.8	5.2	10.9	1462	1308	1703
Total Pore Volume (cm^3^/g)		0.00	0.01	0.01	0.02	0.63	0.62	0.78
Micropore Area (cm^2^/g)		BD *	BD *	BD *	BD *	930	625	941
Micropore Volume (m^3^/g)		BD *	BD *	BD *	BD *	0.46	0.33	0.49
Mesopore + Macropore Volume * (cm^3^/g)		BD *	BD *	BD *	BD *	0.17	0.29	0.29

H-A: Hydrochar where A corresponds to the HTC temperature. SAH-A: Superactivated hydrochar where A corresponds to the HTC temperature. * Sum of mesopore and macropore volume is also termed as V_mame._

**Table 2:** Total and density of oxygen functional groups (acidic) of superactivated hydrochars (SAH180, SAH220 and SAH260).

Adsorbent	Total Oxygen Functional Groups (Acidic) (μmol/g)	Density of Surface Functional Groups (μmol/m^2^)
SAH180	755.827±7.769	0.517±0.005
SAH220	940.420±2.572	0.719±0.002
SAH260	885.837±1.717	0.520±0.001

**Table 3:** Adsorption isotherm model fitting parameters for H_2_, CO_2_, dye and electron storage.

Adsorbate	Model	Temkin			Freundlich			Langmuir		
		R^2^	Model Parameters		R^2^	Model Parameters		R^2^	Model Parameters	
	Adsorbent		k_T_	b_T_		n_F_	k_F_		Q_max_	k_L_
H_2_	H180	0.99681	42.8 ± 4.67	0.113 ± 0.002	0.95993	4.950 ± 0.0170	21.3 ± 0.841	0.93979	37.1 ± 1.1	1.40 ± 0.30
	H220	0.95838	3.91 ± 0.78	0.782 ± 0.005	0.99873	2.618 ± 0.006	12.1 ± 0.210	0.98904	43.4 ± 2.7	0.25 ± 0.05
	H260	0.96633	7.48 ± 2.39	0.067 ± 0.006	0.99813	2.994 ± 0.008	18.7 ± 0.353	0.93140	54.7 ± 4.4	0.35 ± 0.10
CO_2_	H180	0.96703	17.8 ± 3.12	0.015 ± 0.001	0.99839	1.506 ± 0.016	137 ± 1.48	0.99858	255.4 ± 11.6	1.08 ± 0.08
	H220	0.95838	16.6 ± 3.13	0.020 ± 0.002	0.99896	1.420 ± 0.014	100 ± 0.894	0.99871	212.0 ± 11.1	0.86 ± 0.07
	H260	0.96118	17.6 ± 3.21	0.014 ± 0.001	0.99149	1.529 ± 0.035	138 ± 3.26	0.99943	269.2 ± 7.1	1.03 ± 0.05
	H180	N/A	N/A	N/A	0.01300	N/A	N/A	0.99800	588.3	1.42
MB	H220	N/A	N/A	N/A	0.67700	N/A	N/A	0.99800	666.7	0.52
	H260	N/A	N/A	N/A	0.60300	N/A	N/A	0.99900	714.3	0.82
	H180	N/A	N/A	N/A	0.97321	2.319 ± 0.334	69.2 ± 7.33	0.99272	54.1 ± 2.9	9.50 ± 1.37
Electron	H220	N/A	N/A	N/A	0.99351	2.484 ± 0.160	75.2 ± 3.46	0.98912	57.6 ± 3.0	11.79 ± 1.79
	H260	N/A	N/A	N/A	0.98870	2.052 ± 0.170	25.5 ± 1.65	0.99887	21.5 ± 0.5	6.38 ± 0.36

## Data Availability

Not applicable.

## Supplementary Materials

The following supporting information can be downloaded at [110,111,112]: https://www.mdpi.com/article/10.3390/nano12203575/s1, Figure S1: SEM images of loblolly pine biomass, hydrochars (H180, H220, H260) and superactivated hydrochars (SAH180, SAH220, SAH260); Figure S2: CO_2_/N_2_ gas adsorption of superactivated hydrochars (SAH180, SAH220, SAH260); Table S1: Elemental analysis of loblolly pine, hydrochar and superactivated hydrochars, Table S1: Literature comparison for methylene blue dye removal adsorbent from various biomass, Table S3: Literature comparison for electron storage material from various biomass, Table S4: Separation factor of Langmuir Model for MB dye removal in superactivated hydrochars (SAH180, SAH220 and SAH260), Table S5: Langmuir-Freundlich isotherm model fitting parameters for carbon dioxide capture and electron storage.
