# Supplementary material for: Multifunctional Loblolly Pine-Derived Superactivated Hydrochar: Effect of Hydrothermal Carbonization on Hydrogen and Electron Storage with Carbon Dioxide and Dye Removal
Source: Nanomaterials (Basel). 2022 Oct 12;12(20):3575. doi: 10.3390/nano12203575 (PMC9606919; doi:10.3390/nano12203575)
Supplement: Supplementary file 1 [file nanomaterials-12-03575-s001.zip › nanomaterials-1969529-supplementary.pdf]

Supplementary Information

Multifunctional Loblolly Pine-derived Superactivated Hydrochar: Effect of Hydrothermal Carbonization on Hydrogen and Electron Storage with Carbon Dioxide and Dye Removal

Al Ibtida Sultana, Cadianne Chambers, Muzammil M.N. Ahmed, Pavithra Pathirathna and Toufiq Reza*

Department of Biomedical and Chemical Engineering and Sciences, Florida Institute of Technology, Melbourne, FL 32901, USA

* Corresponding Author: email: treza@fit.edu, phone: +1 321 806 6093

Table and Figure Captions

Table S1: Elemental analysis of loblolly pine, hydrochar and superactivated hydrochars

Table S2: Literature comparison for methylene blue dye removal adsorbent from various biomass

Table S3: Literature comparison for electron storage material from various biomass

Table S4: Separation factor of Langmuir Model for MB dye removal in superactivated hydrochars (SAH180, SAH220 and SAH260).

Table S5: Langmuir-Freundlich isotherm model fitting parameters for carbon dioxide capture and electron storage.

Figure S1: SEM images of loblolly pine biomass, hydrochars (H180, H220, H260) and superactivated hydrochars (SAH180, SAH220, SAH260)

Figure S2: CO_2/N_2 gas adsorption of superactivated hydrochars (SAH180, SAH220, SAH260)

Table S1: Elemental analysis of loblolly pine, hydrochar and superactivated hydrochars

Material	Mass Yield (%)	Ash (%)	N (%)	C (%)	H (%)	O (%)
LP	100.0	1.59	4.38	46.21	5.80	42.02
H180	77.6	0.37	3.42	48.67	4.90	42.62
H220	65.4	0.36	3.75	50.95	4.20	40.76
H260	47.3	3.36	3.10	58.43	3.50	31.65
SAH180	11.8	6.40	3.53	77.07	0.06	12.93
SAH220	13.1	8.10	3.93	82.20	0.07	5.70
SAH260	17.1	9.84	3.01	84.13	0.02	3.00

Table S2: Literature comparison for methylene blue dye removal adsorbent from various biomass

Biomass Precursor	BET SSA (m^2/g)	Q_{max} (mg/g)	Adsorption Isotherm Model	References
Loblolly Pine	1703	719.4	Langmuir	*This work*
Chitosan	3496	890.3	Langmuir	[1]
Spherical Carbon	1534	704.2	Redlich-Peterson	[2]
Sewage Sludge + Coconut Shell	873.5	623.4	Langmuir	[3]
Pentace species sawdust	914.2	357.1	Redlich-Peterson	[4]
Bamboo chip	720.7	305.3	Freundlich	[5]
Dragon fruit peel	756.3	195.2	Langmuir	[6]
Wood	167.7	59.9	-	[7]

Table S3: Literature comparison for electron storage material from various biomass

**Biomass Precursor**	**Mass Loading (mg)**	**BET SSA (m^2/g)**	**Specific Capacitance (F/g)**	**Current Density (A/g)**	**References**
Loblolly Pine	0.18	1462	47.23	0.1	*This work*
Mung bean husk	2	2131	390	1	[8]
Lignin	10	2486	384	0.04	[9]
Xylose	5	3103	340	0.5	[10]
Lignin	3	1504	324	0.5	[11]
Green needle coke	2	807.7	274.9	1	[12]
Corn straw	2	1781	98	1	[13]

Table S4: Separation factor of Langmuir Model for MB dye removal in superactivated hydrochars (SAH180, SAH220 and SAH260).

**Initial Conc. (ppm)**	**SAH 180**	**SAH 220**	**SAH 260**
700	0.0010	0.0019	0.0012
800	0.0009	0.0017	0.0011
900	0.0008	0.0015	0.0010
1000	0.0007	0.0013	0.0009
1100	0.0006	0.0012	0.0008
1200	0.0006	0.0011	0.0007

Table S5: Langmuir-Freundlich isotherm model fitting parameters for carbon dioxide capture and electron storage.

Adsorbate	Sample ID	Langmuir-Freundlich Model			
		F_{mLF}	K_{LF}	β	R^2
Electron	SAH180	43.4 ± 1.5	13.92 ± 0.78	1.55 ± 0.14	0.99834
	SAH220	80.0 ± 50.2	4.97 ± 9.19	0.70 ± 0.33	0.99073
	SAH260	19.7 ± 1.2	7.65 ± 0.93	1.11 ± 0.09	0.99900
CO_2	SAH180	345.3 ± 13.8	0.45 ± 0.04	0.80 ± 0.14	0.99994
	SAH220	308.2 ± 15.9	0.33 ± 0.04	0.82 ± 0.93	0.99995
	SAH260	271.2 ± 15.3	1.00 ± 0.19	0.90 ± 0.06	0.99932

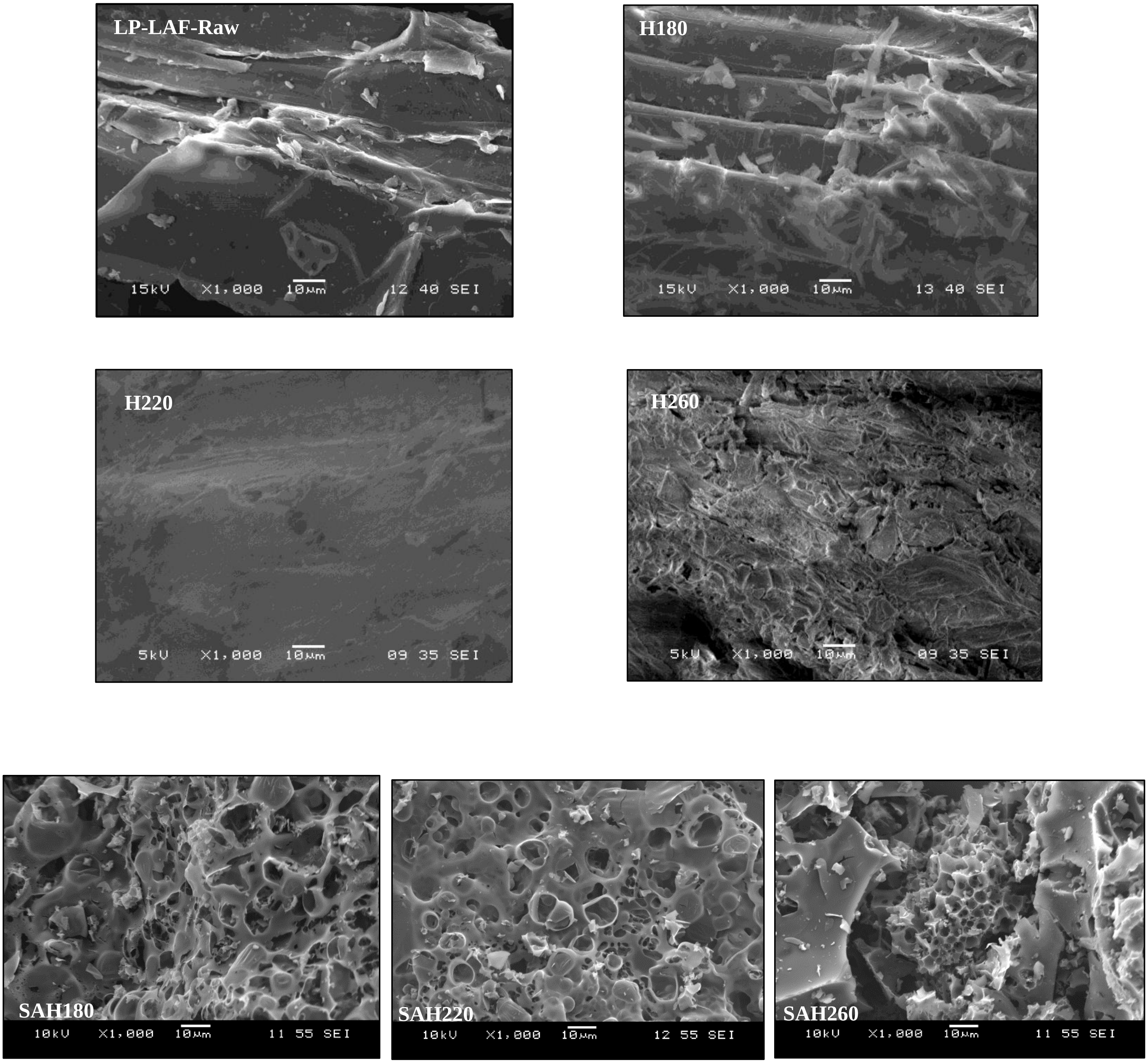

Figure S1: SEM images of loblolly pine biomass, hydrochars (H180, H220, H260) and superactivated hydrochars (SAH180, SAH220, SAH260)

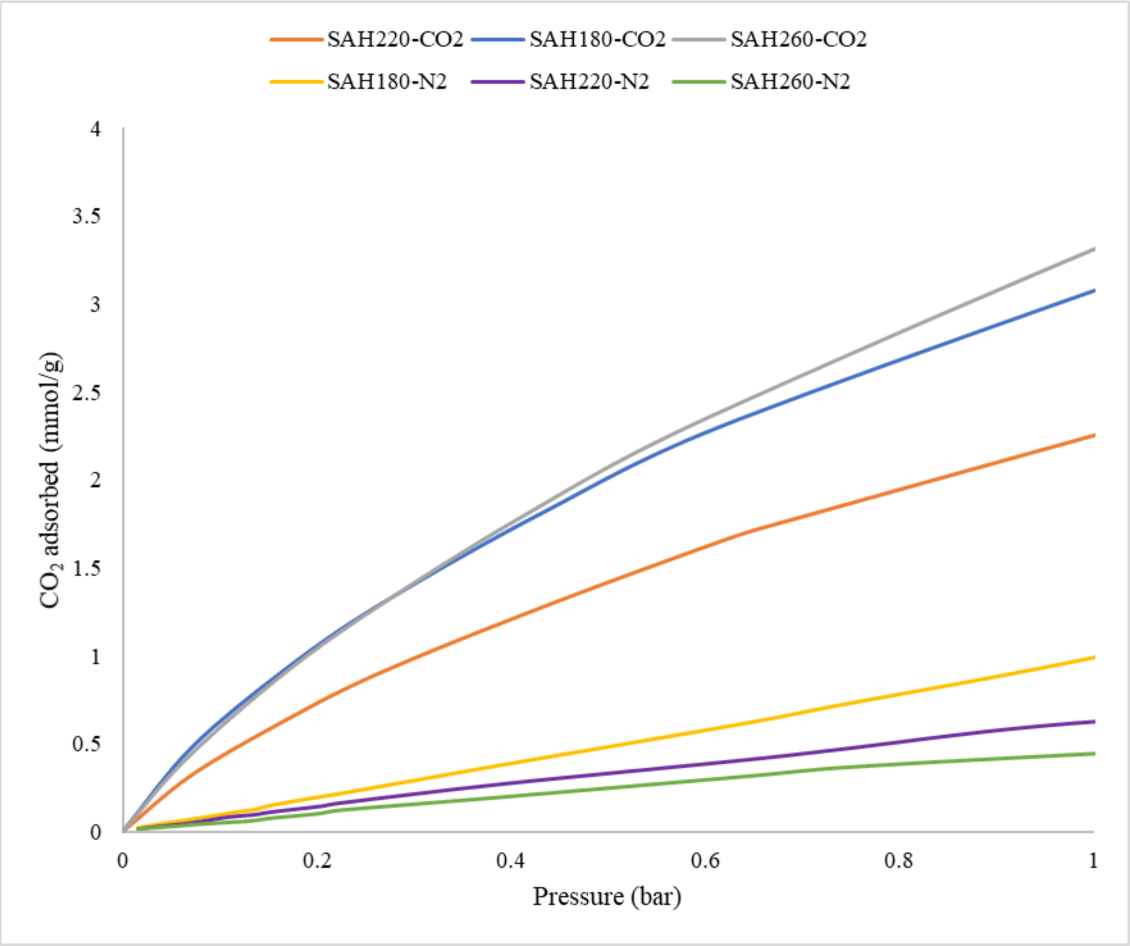

Figure S2: CO_2/N_2 gas adsorption of superactivated hydrochars (SAH180, SAH220, SAH260)

References

1. Jin, Q.; Li, Y.; Yang, D.; Cui, J. Chitosan-Derived Three-Dimensional Porous Carbon for Fast Removal of Methylene Blue from Wastewater. *RSC Adv.* **2018**, *8*, 1255–1264, doi:10.1039/C7RA11770A.
2. Bedin, K.C.; Martins, A.C.; Cazetta, A.L.; Pezoti, O.; Almeida, V.C. KOH-Activated Carbon Prepared from Sucrose Spherical Carbon: Adsorption Equilibrium, Kinetic and Thermodynamic Studies for Methylene Blue Removal. *Chemical Engineering Journal* **2016**, *286*, 476–484, doi:10.1016/j.cej.2015.10.099.
3. Tu, W.; Liu, Y.; Xie, Z.; Chen, M.; Ma, L.; Du, G.; Zhu, M. A Novel Activation-Hydrochar via Hydrothermal Carbonization and KOH Activation of Sewage Sludge and Coconut Shell for Biomass Wastes: Preparation, Characterization and Adsorption Properties. *Journal of Colloid and Interface Science* **2021**, *593*, 390–407, doi:10.1016/j.jcis.2021.02.133.
4. Khasri, A.; Bello, O.S.; Ahmad, M.A. Mesoporous Activated Carbon from Pentace Species Sawdust via Microwave-Induced KOH Activation: Optimization and Methylene Blue Adsorption. *Res Chem Intermed* **2018**, *44*, 5737–5757, doi:10.1007/s11164-018-3452-7.
5. Jawad, A.H.; Abdulhameed, A.S. Statistical Modeling of Methylene Blue Dye Adsorption by High Surface Area Mesoporous Activated Carbon from Bamboo Chip Using KOH-Assisted Thermal Activation. *Energ. Ecol. Environ.* **2020**, *5*, 456–469, doi:10.1007/s40974-020-00177-z.
6. Jawad, A.H.; Saud Abdulhameed, A.; Wilson, L.D.; Syed-Hassan, S.S.A.; ALOthman, Z.A.; Rizwan Khan, M. High Surface Area and Mesoporous Activated Carbon from KOH-Activated Dragon Fruit Peels for Methylene Blue Dye Adsorption: Optimization and Mechanism Study. *Chinese Journal of Chemical Engineering* **2021**, *32*, 281–290, doi:10.1016/j.cjche.2020.09.070.
7. Danish, M.; Ahmad, T.; Hashim, R.; Said, N.; Akhtar, M.N.; Mohamad-Saleh, J.; Sulaiman, O. Comparison of Surface Properties of Wood Biomass Activated Carbons and Their Application against Rhodamine B and Methylene Blue Dye. *Surfaces and Interfaces* **2018**, *11*, 1–13, doi:10.1016/j.surfin.2018.02.001.
8. Song, M.; Zhou, Y.; Ren, X.; Wan, J.; Du, Y.; Wu, G.; Ma, F. Biowaste-Based Porous Carbon for Supercapacitor: The Influence of Preparation Processes on Structure and Performance. *Journal of Colloid and Interface Science* **2019**, *535*, 276–286, doi:10.1016/j.jcis.2018.09.055.
9. Wu, Y.; Cao, J.-P.; Zhao, X.-Y.; Zhuang, Q.-Q.; Zhou, Z.; Huang, Y.; Wei, X.-Y. High-Performance Electrode Material for Electric Double-Layer Capacitor Based on Hydrothermal Pre-Treatment of Lignin by ZnCl2. *Applied Surface Science* **2020**, *508*, 144536, doi:10.1016/j.apsusc.2019.144536.
10. Sun, W.; Zhang, Y.; Yang, Z.; Yang, F. High-performance Activated Carbons for Electrochemical Double Layer Capacitors: Effects of Morphology and Porous Structures. *Int J Energy Res* **2020**, *44*, 1930–1950, doi:10.1002/er.5047.
11. Li, H.; Shi, F.; An, Q.; Zhai, S.; Wang, K.; Tong, Y. Three-Dimensional Hierarchical Porous Carbon Derived from Lignin for Supercapacitors: Insight into the Hydrothermal Carbonization and Activation. *International Journal of Biological Macromolecules* **2021**, *166*, 923–933, doi:10.1016/j.ijbiomac.2020.10.249.
12. Cheng, J.; Lu, Z.; Zhao, X.; Chen, X.; Liu, Y. Green Needle Coke-Derived Porous Carbon for High-Performance Symmetric Supercapacitor. *Journal of Power Sources* **2021**, *494*, 229770, doi:10.1016/j.jpowsour.2021.229770.
13. Liu, D.; Wang, Y.; Jia, B.; Wei, J.; Liu, C.; Zhu, J.; Tang, S.; Wu, Z.; Chen, G. Microwave-Assisted Hydrothermal Preparation of Corn Straw Hydrochar as Supercapacitor Electrode Materials. *ACS Omega* **2020**, *5*, 26084–26093, doi:10.1021/acsomega.0c03605.
